# Development of an algorithm for biomedical image analysis of the spatial organization of collagen in breast cancer tissue of patients with different clinical status

**DOI:** 10.1002/2211-5463.13773

**Published:** 2024-02-21

**Authors:** Nataliia Lukianova, Oleksandr Mushii, Taras Zadvornyi, Vasyl Chekhun

**Affiliations:** ^1^ R.E. Kavetsky Institute of Experimental Pathology, Oncology and Radiobiology NAS of Ukraine Kyiv Ukraine

**Keywords:** breast cancer, collagen, microphoto analysis, spatial organization, tumor progression, tumor‐stroma ratio

## Abstract

Collagen, the main component of the tumor microenvironment, plays a key role in the development of breast cancer (BCa); however, the specific changes in its spatial organization during tumor progression have not been definitively elucidated. The existing and available methods for assessing the morphometric parameters of the stroma's fibrous component are insufficient for a detailed description of the state of collagen fibers and for assessing its changes to evaluate the aggressiveness of the BCa course. The aim of the work was to develop an algorithm for microphoto analysis to assess the spatial organization of collagen in BCa tissue of patients with different clinical statuses. The study was conducted on 60 tissue samples of stage I‐II BCa. The processed images were analyzed using the software packages CurveAlign v4.0 and imagej. We established that the increase in BCa stage and the decrease in tumor differentiation grade are associated with decreased length, width, and straightness of collagen fibers, as well as their increased density. The formation of an aggressive basal molecular BCa subtype was accompanied by an increase in tumor‐stroma ratio. The obtained results indicate the possibility of practical application of the developed algorithm for evaluating the spatial organization of collagen in BCa tissue to predict the aggressiveness of the disease course.

## Graphical abstract

In order to assess the condition of the tumor collagen matrix, we developed an algorithm for microphoto analysis, which uses Malory's trichrome staining, and also involves specialized software packages (curve align and imagej) for morphometric studies. After completing the analysis, we obtained morphometric indicators of the mammary gland malignant neoplasms stromal fibrous components.

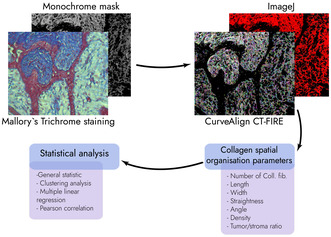

**Abbreviations**
BCa: breast cancer
SHG: second harmony generation
TSR: tumor stroma ratio

Presently, breast cancer (BCa) is the most common tumor type among women. About 2.25 million new BCa cases are diagnosed annually in the world. It is predicted that in the next 20 years, the incidence of BCa will increase by 1.5 times, and the mortality rate will double [1].

A key role in the mechanisms of cancer progression, including BCa, belongs to the tumor microenvironment, alterations that are directly reflected in the components of the tumor lesion [2]. Changes in stromal tumor‐associated cells at the epigenetic level are directly reflected in the altered expression of most growth factors and drivers of carcinogenesis in tumor cells [3]. The components of the tumor microenvironment, in particular the immune infiltrate and fibroblasts, stimulate and form the metastatic niche [4, 5].

The non‐cellular elements of the tumor microenvironment are the least studied agents of tumor progression; they include collagen, which forms a dense three‐dimensional network of interwoven fibrous structures and is the most abundant fibrillar protein in the body. It was established that BCa progression is accompanied by a change in collagen content and increased fibrosis in the tumor lesion, which correlates with the activation of the HIF‐1 and TGF‐β signaling cascades [6].

It has been shown that in malignant neoplasms of the mammary gland changes in the spatial organization of collagen fibers and an increase in entropy in the stromal microenvironment could be observed [7]. The results of other studies indicated that during tumor progression in the peritumoral zone, uniform alignment and straightening of fibers relative to each other was observed, which may indicate the facilitation of tumor cell invasion into surrounding tissues and stimulation of metastasis [8]. Despite all the above, there is still no unified panel of characteristics of the fibrous element in the composition of the microenvironment, which could be used to assess the risk of cancer progression and improve the prognosis.

A classic method of examining the state of the stroma using light microscopy is staining sections of tumor tissue with hematoxylin and eosin. However, the study of individual fibers in the stromal component requires the use of more complex staining techniques and specific analysis of the obtained histological microphotographs. Today, research on morphometric parameters is mainly carried out using the second harmonic generation (SHG) microscopy. The basis of this method is the capture by a microscope of the so‐called second harmonic light waves, which arise when a tissue section is exposed to a light source [9]. Another method of visualizing collagen in the composition of tumor tissue is the use of polarization microscopy. To enhance the contrast of the image, sections can be stained with picrosirius red [10]. However, the methods described require high‐cost reagents, the availability of expensive equipment and qualified personnel. Taking into account the above, the aim of the work was to develop an algorithm for microphoto analysis to assess the spatial organization of collagen in BCa tissue of patients with different clinical statuses.

## Materials and methods

The BCa tissue samples (*n* = 60) were obtained from patients with breast cancer who were treated at the Kyiv Oncology Center between 2013 and 2015. The work was approved by the Institutional Review Board and Research Ethics Committee of R.E. Kavetsky Institute of Experimental Pathology, Oncology and Radiobiology of the National Academy of Sciences of Ukraine (protocol №3 dated October 12, 2023) and was conducted following the Declaration of Helsinki and Good Clinical Practice guidelines. The clinical and pathological characteristics of the studied BCa cases are described in more detail in Table 1. All patients gave informed written consent for the use of clinical data for scientific purposes. Patients were not prescribed neoadjuvant polychemotherapy. The stage of the tumor process was determined according to the International TNM Classification [11]. The average age of the patients was 63.2 ± 7.6 years and ranged from 28 to 89 years.

**Table 1:** General patients' clinical characteristics.

Tumor size		Lymph node involvement		Tumor grade		Molecular subtype	
T1	T2	N0	N1	G2	G3	Luminal	Basal/HER2 pos.
30	30	41	19	44	16	43	17

Determination of collagen in the composition of the tumor stroma was carried out using the histochemical staining method by Mallory (Mallory trichrome; DiaPath, Martinengo, Italy) according to the manufacturer's recommendations. Microscopy of histological micro‐preparations was carried out using the optical system AxioScope A1 (Carl Zeiss, Oberkochen, Germany). Photos were taken using an AxioCam ICc5 camera (Carl Zeiss) at magnification ×260.

Adobe Photoshop CC (Adobe Systems Inc., San Jose, California, USA) was used to process microphotographs before direct analysis. The analysis of the morphometric characteristics of the spatial organization of collagen fibers was carried out using the software curvealign v. 4.0. beta (https://loci.wisc.edu/curvealign/) and imagej (https://imagej.nih.gov/ij/).

Statistical analysis was performed using the graphpad prism v.8.0 (GraphPad Software Inc., USA) and statistica 12.0 (StatSoft, USA) programs. The Mann–Whitney *U*‐test was used to assess statistical significance when comparing the groups. Clustering analysis was performed, using the k‐means method. The correlation matrix was calculated by Pearson. A difference was considered significant at *P* < 0.05.

### Algorithm for microphoto analysis for evaluating the spatial organization of collagen in BCa tissue

**Fig. 1:**
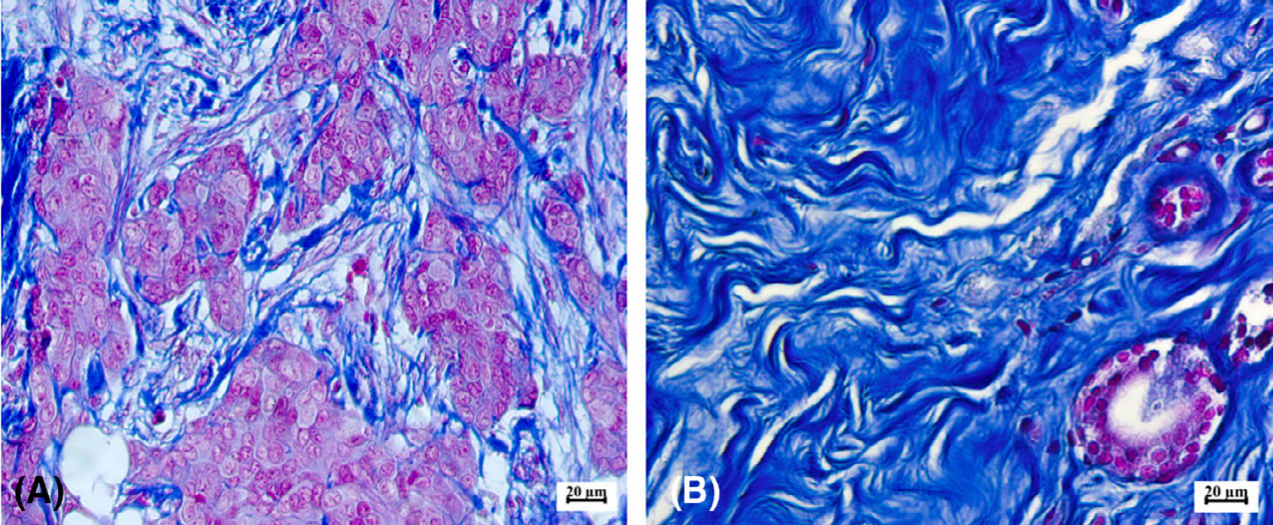
Breast cancer tissue, Trichrome Mallory staining (260×), scale bar – 20 μm. Stromal tissue, enriched with a fibrillar component, is colored blue, tumor parenchyma is magenta. (A) tumor tissue with a low content of fibrillary stroma, (B) tumor tissue with a high content of fibrillary stroma.

**Fig. 2:**
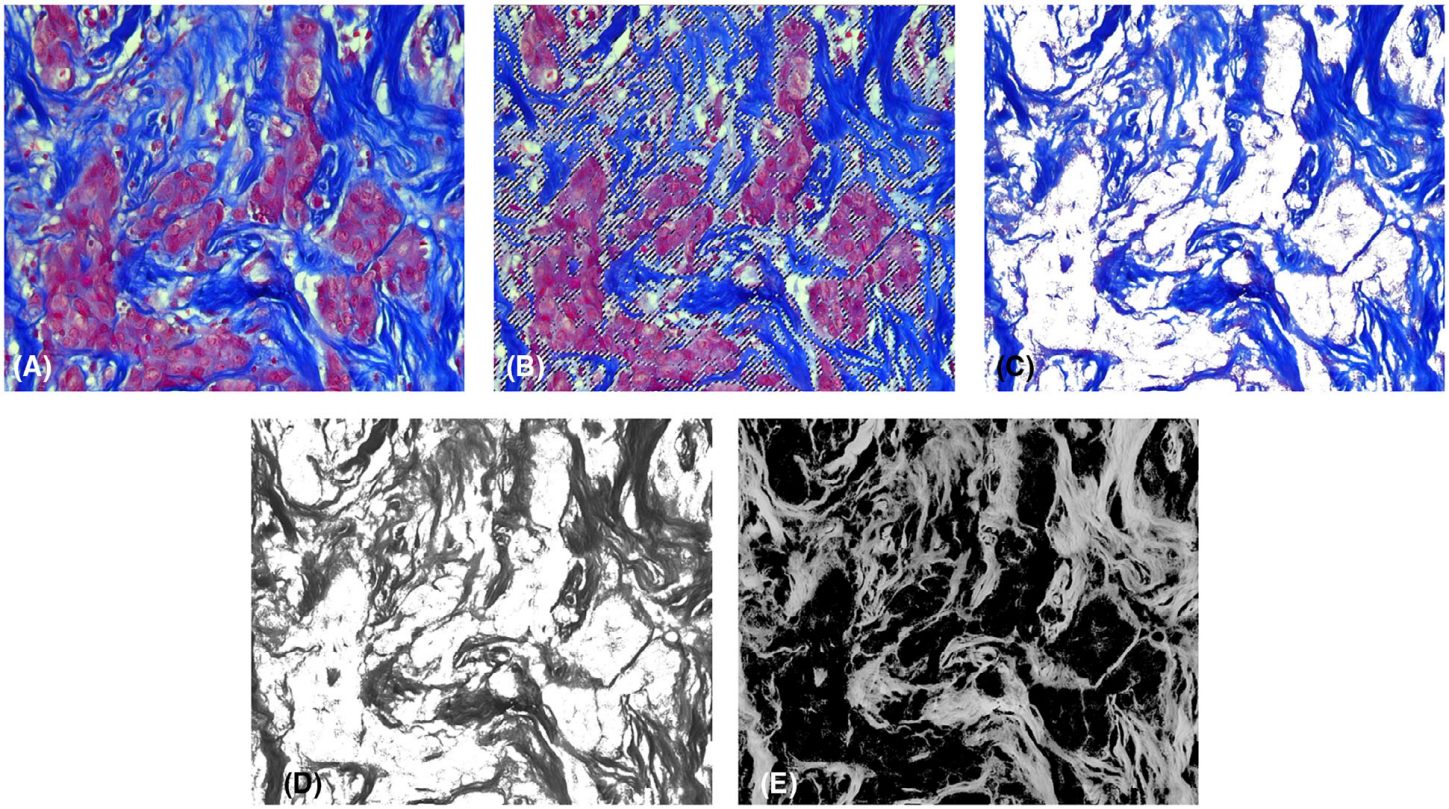
Adobe Photoshop image editing algorithm. (A) raw image with no applications; (B) stroma segregation by using magic wand tool; (C) separated stromal component; (D) black‐and‐white monochrome mask; (E) inverted black‐and‐white monochrome mask.

**Fig. 3:**
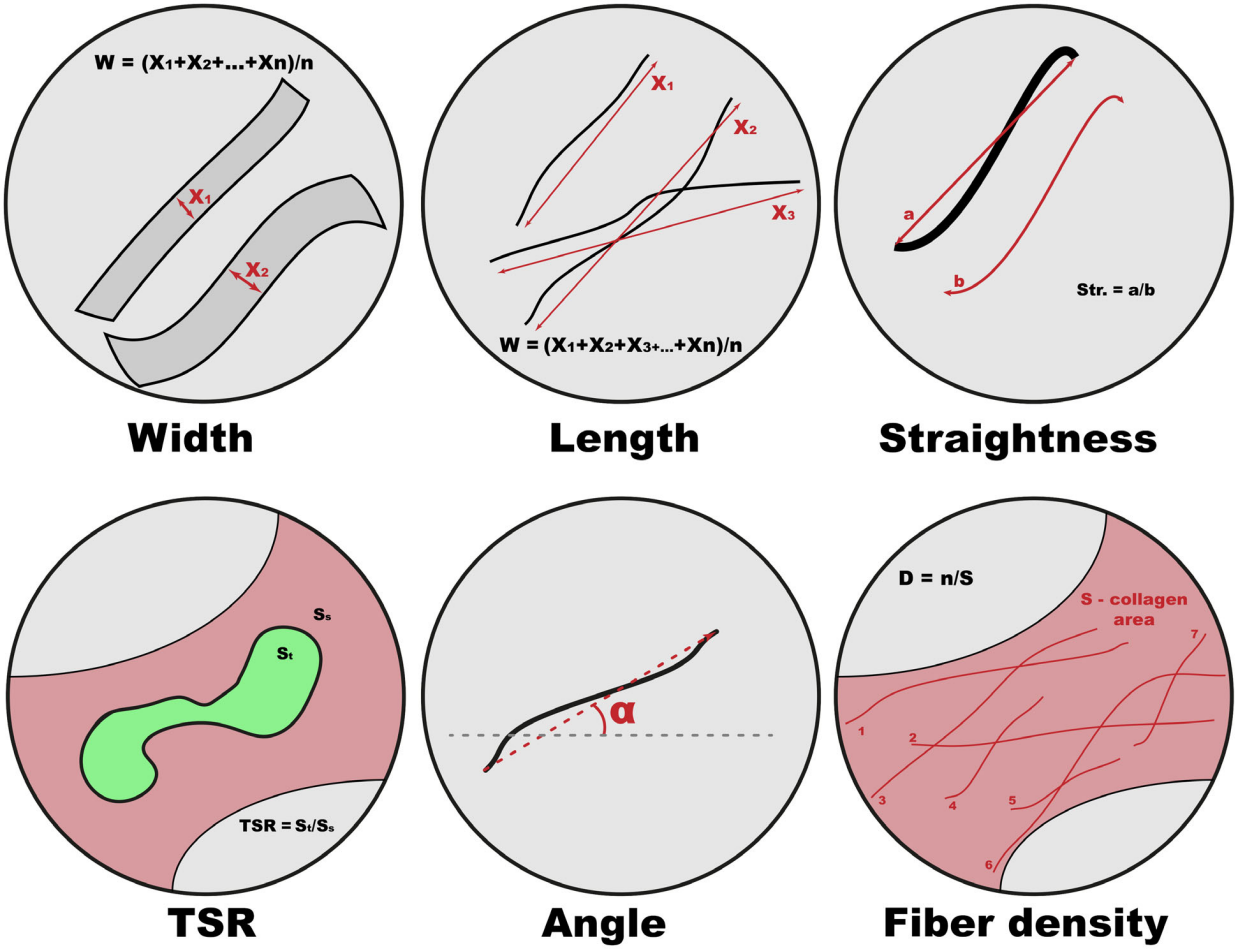
All parameters of collagen fibers' spatial organization and tumor stroma status measured and estimated in the research.

**Fig. 4:**
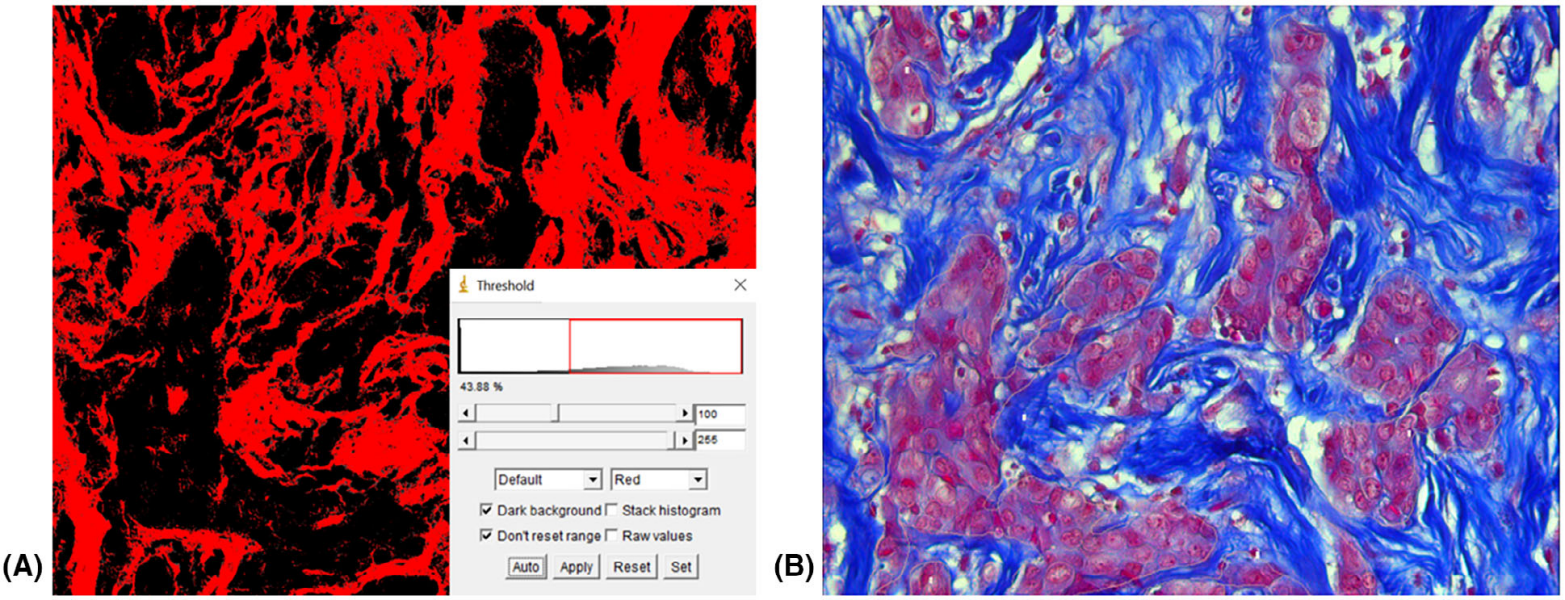
Measuring of stromal and tumor areas in imagej for TSR and fiber density calculation. (A) Stromal area measured by Threshold tool; (B) tumor area measured in classic imagej algorithm.

1. To obtain histological preparations, serial sections of 5 μm thickness were made from paraffin‐embedded tumor samples. Subsequently, the tissue was stained by the histochemical method of Mallory. This type of staining is used to study the fibrous structures of connective tissue. The method is based on the unique property of aniline blue to stain collagen fibers in greenish‐blue color, and acid fuchsin to stain elastic fibers in red. As a result of such a staining, collagen fibers are stained dark blue, nuclei, elastin fibers—red, amyloid, hyaline—blue, muscle tissue—orange, neuroglia—red‐violet, and erythrocytes—yellow (Fig. 1). Taking into account the specificity of the morphological structure of the tumor tissue, the method allows the separation of the fibrous collagen component of the neoplasm from other tissue elements with high efficiency, which was then used for the segregation of fibrous elements in the images.
2. Microphotographs of the preparations were obtained using a digital microscopy complex—AxioScope A1 (Carl Zeiss) and an AxioCam ICc5 camera (Carl Zeiss) with a magnification of ×260. All microphotographs were saved in .*jpeg* format, the image size was 2452×2056 pixels.
3. The key element of the developed algorithm was the processing of microphotographs using the Adobe Photoshop CC graphic editor (Fig. 2), which was used to separate the elements of the collagen framework stained in dark blue and other components of the tumor tissue in red and yellow. With the help of a smart object selection algorithm using the magic wand tool, which selects pixels based on tone and color, the fibrous framework of the tumor was separated. In the parameters of the tool, the optimal tolerance value was chosen—the tolerance deviation of the pixel tone from the given reference value on the micrograph. After selecting the area of the microphotograph with the non‐cellular, stromal component, it was cut out on a new layer with a white background. Next, the resulting image was converted to monochromatic mode, and the black‐and‐white image was inverted. The last step was necessary for the following use of the obtained images in a specialized algorithm of analysis.
4. Direct analysis of the spatial organization of collagen was performed using the curvealign software (Madison, Wisconsin, USA). This software package allows to analysis of images obtained via SHG microscopy. The algorithm proposed by us allows the use of the curvealign software package for the analysis of images obtained using light microscopy, which leads to a reduction in cost and unification of the technique of researching fibrous elements of different tumor types. We obtained the values of the length, width, straightness, and number of the fibers, as well as their angles to the regions of tumor growth (Fig. 3). It should be mentioned, that such parameters as the length and width of collagen fibers, obtained during analysis in the CT‐FIRE mode, are expressed in pixels and do not depend on the size of the obtained image. However, to translate the length and width values into μm dimensions, we took into account the microphoto expansion (number of pixels per unit area). Both parameters make it possible to evaluate the functional state of the tissue, namely: its density, strength, and elasticity. The straightness (“Straightness”) of the fibers is presented as a coefficient ranging from 0 to 1, where the value directly correlates with the degree of straightness of the fibers. As for the “Angle” indicator, its value indicates the size of the angle formed by each fiber at the intersection with the nearest layer of epithelial cells. In general, this group of indicators makes it possible to evaluate the peculiarities of the spatial organization of collagen fibrils, their orientation, orderliness, and level of anisotropy.
5. To calculate tumor‐stroma ratio (TSR) and fiber density, the areas of stroma and tumor elements were additionally measured using imagej. The area of tumor cells was calculated in the classic mode. Black‐and‐white microphotograph masks were used to calculate the stromal area, as described in Step 3. The inverted monochromatic masks were loaded into the imagej program and the area of regions stained in gray was measured using the Threshold tool automatically (Fig. 4).
6. At the next stage, calculations and conversion of raw data were carried out. The CurveAlign module outputs the results as coefficients or dimensions in pixels. For the convenience of evaluating the obtained indices and unifying the measurement units, the length and width values were converted into micrometers. The TSR ratio was measured by dividing the area of the tumor component in the tissue by the area of the stroma. Fiber density per unit area was calculated by dividing the stromal area by the number of fibers within the microphotograph. After that, all values of morphometric characteristics of collagen organization were ready for statistical analysis

## Results

**Fig. 5:**
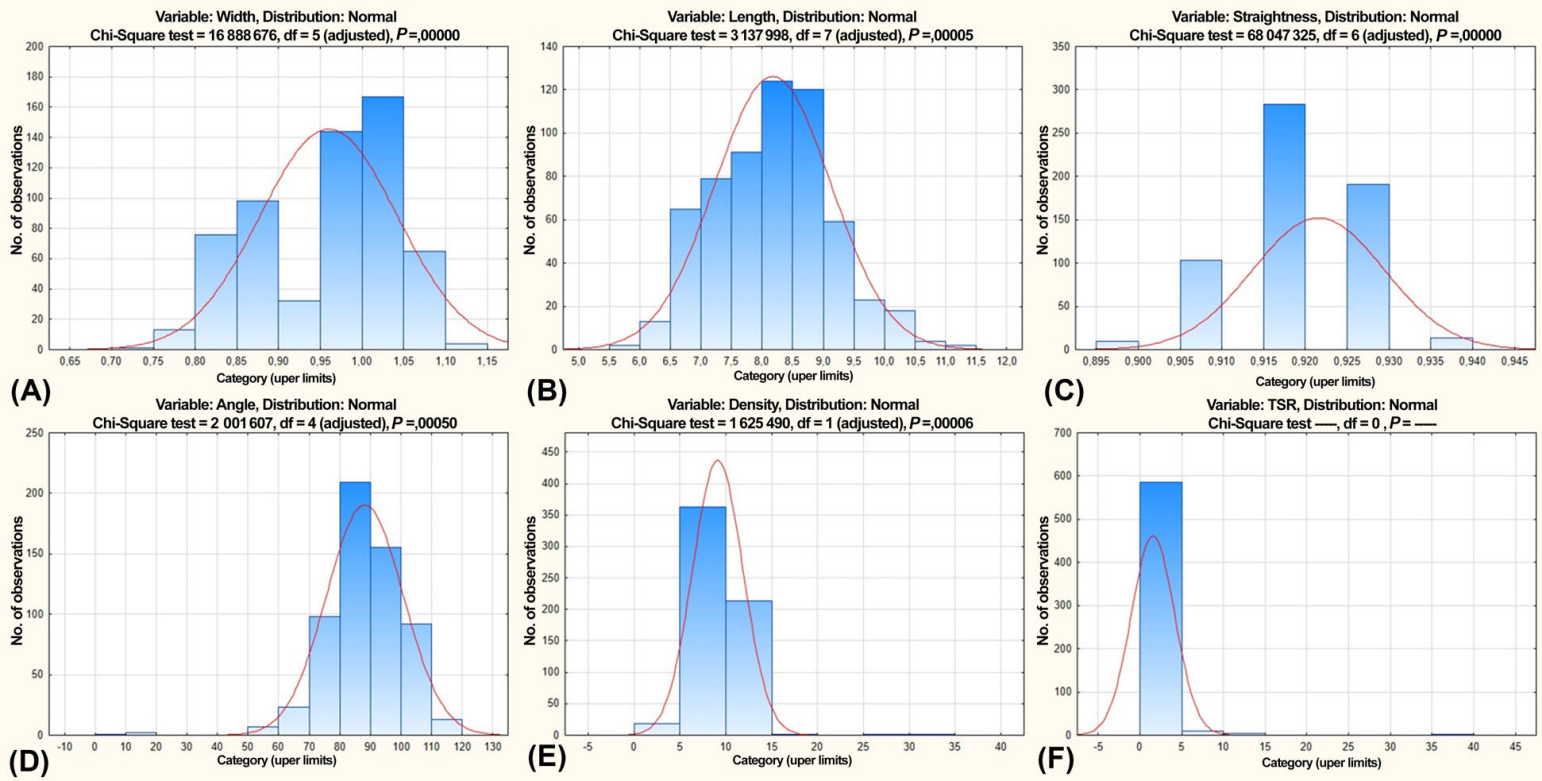
curve align data distribution plot: (A) width; (B) length; (C) straightness; (D) angle; (E) density; (F) TSR.

Tumor tissue samples of 60 patients with breast cancer were used for the study. Morphometric analysis was performed on pre‐processed photomicrographs of Malory‐stained breast cancer tissue. For this purpose, microphotographs in 10 fields of view were taken from each micropreparation of BCa tissue stained according to Malory. Analyzing the quantitative characteristics of the spatial collagen organization, the parameters of about 700 000 individual fibers in the composition of the tumor stroma were studied. The data distribution is presented in Fig. 5.

**Fig. 6:**
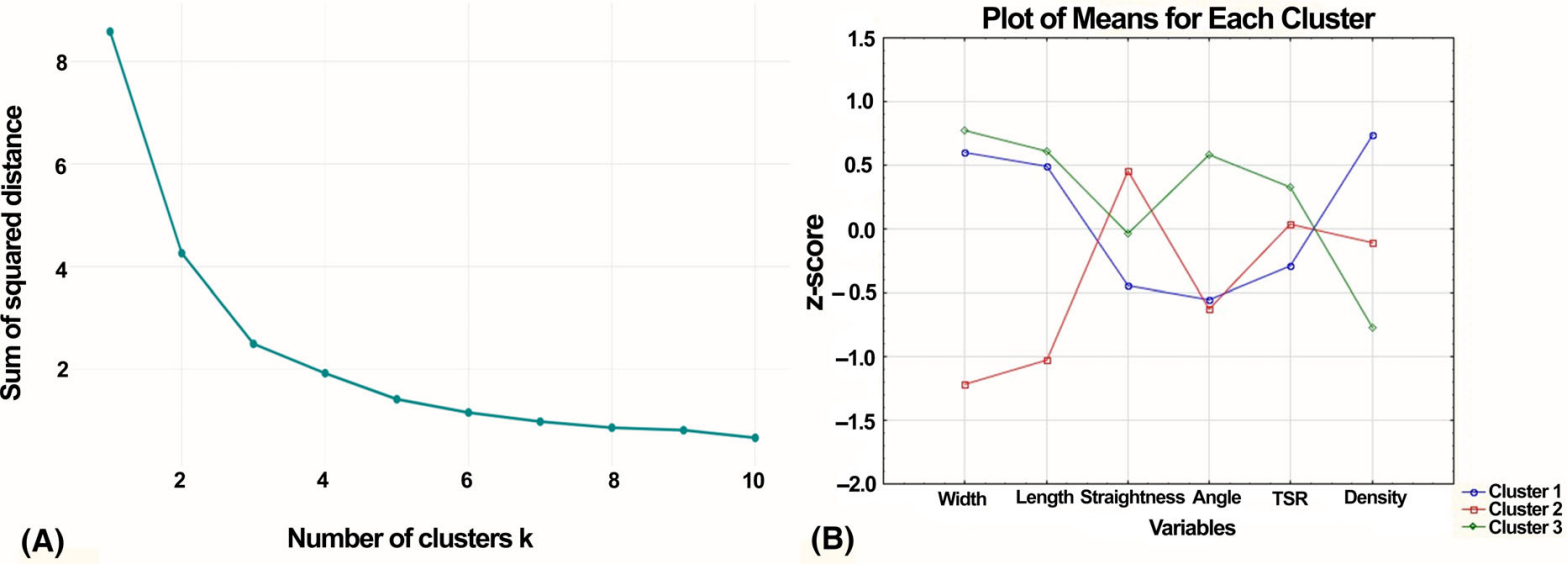
Cluster analysis: (A) elbow diagram to determine the number of clusters, (B) graph of mean values of morphometric parameters of collagen spatial organization for individual clusters.

We performed cluster analysis using the k‐means method, as a result of which all photomicrographs were divided into 3 clusters depending on the combination of morphometric characteristics (length, width, straightness, angle, density, and TSR). To plot the average values of the morphometric parameters we studied within each cluster, the values were standardized using the Statistica 12 program (Fig. 6, Table 2).

**Table 2:** Euclidean distances between clusters.

Euclidean distances			
	Cluster 1	Cluster 2	Cluster 3
Cluster 1	0.000000	1.206437	0.691249
Cluster 2	1.098379	0.000000	1.475593
Cluster 3	0.831414	1.214740	0.000000

Subsequently, the obtained clusters were analyzed according to clinical features, as well as according to the morphometric characteristics of the spatial organization of collagen. As can be seen from the data given in the Table 3, cluster 1 included BCa samples of both investigated categories T (T1 and T2–59.6% and 40.4% of cases, respectively) and N (N0 and N1 32.2% and 67.8%, respectively) according to the TNM classification. The second cluster contained BCa samples from patients mainly with categories T2 and N0 (90% and 91.6% of cases, respectively). In contrast to this, cluster 3 consisted of BCa samples mainly from patients with small neoplasms (80.0%) with metastatic lesions of regional lymph nodes (90%). The majority of BCa samples in all studied clusters had a moderate degree of differentiation (Table 3). It should be noted that clusters 1 and 2 were represented by samples mainly of neoplasms of luminal A and B subtypes, while cluster 3 included cases of HER2/neu positive and basal molecular subtypes of BCa (Table 3).

**Table 3:** Each cluster's general patients' clinical characteristics.

Characteristic	Tumor size (T)		Lymph node involvement		Differentiation grade (G)		Molecular subtype	
	T1	T2	N0	N1	G2	G3	LUM	BAS
Cluster 1								
%	59.6	40.4	32.2	67.8	53.9	46.1	98.26	1.74
N. photos	137	93	74	156	124	106	226	4
Cluster 2								
%	10	90	91.6	8.4	93.2	6.8	100	0
N. photos	19	171	174	16	177	13	190	0
Cluster 3								
%	80	20	90	10	78.3	21.7	7.8	91.2
N. photos	144	36	162	18	141	39	14	166

When analyzing the collagen fibers spatial characteristics, it was established that samples of cluster 1 were characterized by the highest indicators of the density (10.16149 ± 0.123968 fibers/100 μm^2^), as well as low indicators of straightness (0.92052 ± 0.003632 a.u.) and TSR (1.03380 ± 1.451433 a.u.) in comparison with similar indicators of clusters 2 and 3 (Table 4). The BCa samples from cluster 2 had the highest values of straightness (0.92339 ± 0.004933 a.u.), as well as the smallest among the 3 studied clusters' indicators of length (7.94594 ± 0.772611 μm), width (0.93461 ± 0.076355 μm) and angle (81.17072 ± 1.761953°) (Table 4). A characteristic feature of breast cancer neoplasms included in cluster 3 were significantly greater indicators of length (8.42019 ± 0.798403 μm), and fiber width (0.99823 ± 0.061393 μm), angle (92.13478 ± 1.821311°), TSR (1.91686 ± 0.628126 a.u.) as well as the lowest fiber density (9.11197 ± 0.502637 fibers/100 μm^2^) (Table 4).

**Table 4:** Quantitative indicators of the collagen spatial organization in BCa tissue of each cluster.

	Cluster 1		Cluster 2		Cluster 3	
	Mean	SD	Mean	SD	Mean	SD
Width	0.94605	0.079657	0.93461	0.076355	0.99823	0.061393
Length	8.09324	0.691478	7.94594	0.772611	8.42019	0.798403
Straightness	0.92052	0.003632	0.92339	0.004933	0.92181	0.004967
Angle	86.46778	1.548299	81.17072	1.761953	92.13478	1.821311
TSR	1.03380	0.451433	1.36305	0.628126	1.91686	0.790966
Density	10.16149	0.123968	9.30969	0.837603	9.11197	0.502637

The intensity of the colour filling depends on the mean values of the morphometric parameters among the identified clusters.

When analyzing the relationship between the morphometric parameters of collagen fibers in BCa tissue and the clinical and pathological features of the tumor process, a significant decrease in the length of the fibers was established with increasing tumor size (T) (*P* = 0.0006) and a decrease in the tumor differentiation grade (*P* < 0.0001), as well as the presence of metastatic lesions in regional lymph nodes (N) (*P* < 0.0001). Similar changes were observed when studying the thickness of collagen fibers. The smallest width of collagen fibers was noted in neoplasms of small size (T1) (*P* < 0.0001), in cases of category N1 (*P* < 0.0001), and in tumors of low differentiation grade (*P* = 0.0042).

**Fig. 7:**
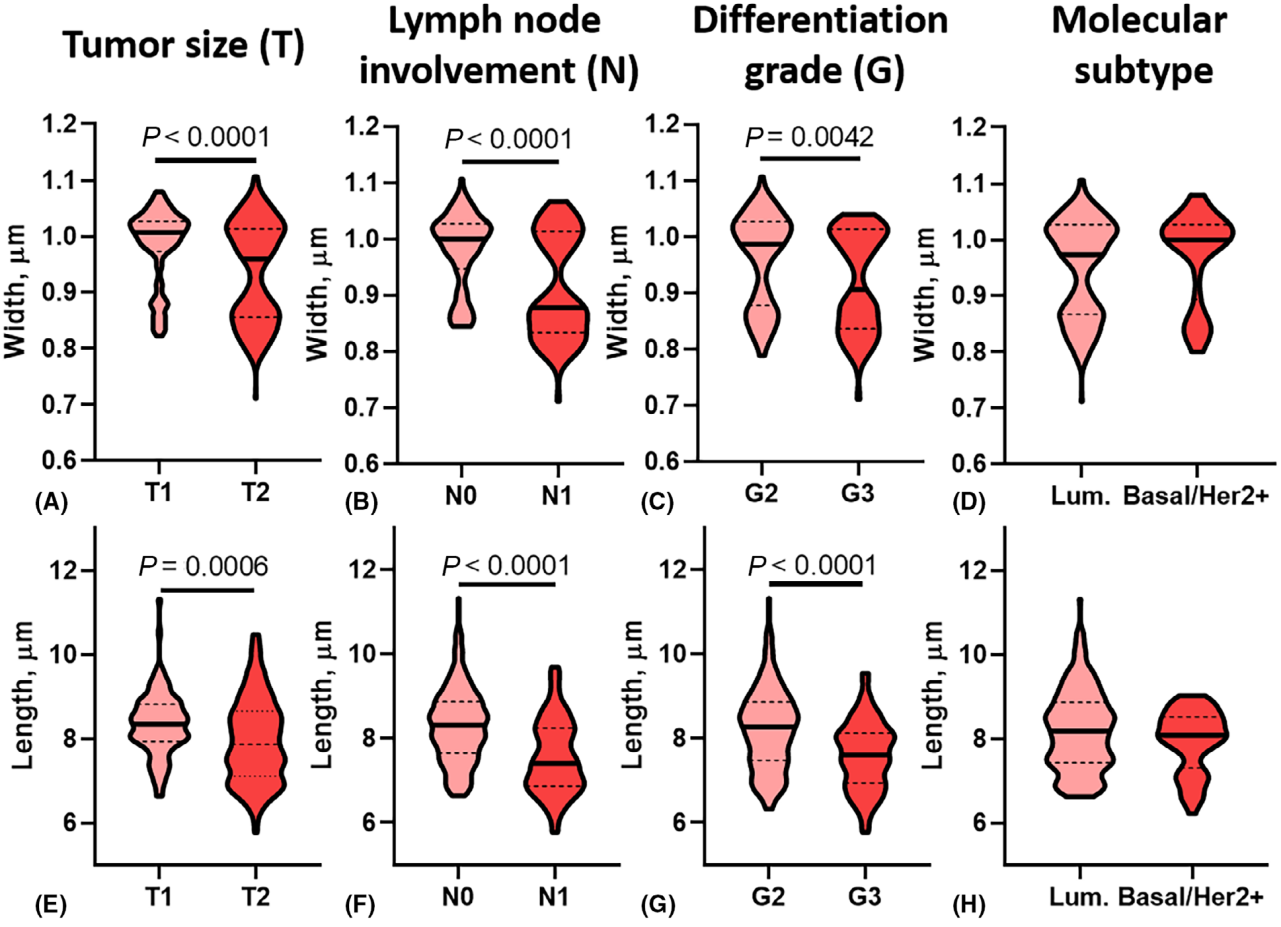
Quantitative indicators (A–D – width, E–H – length) of the spatial organization of collagen in BCa tissue. Lum – luminal molecular subtype; Basal/Her2+ − Her2/neu positive or basal molecular subtype group.

**Fig. 8:**
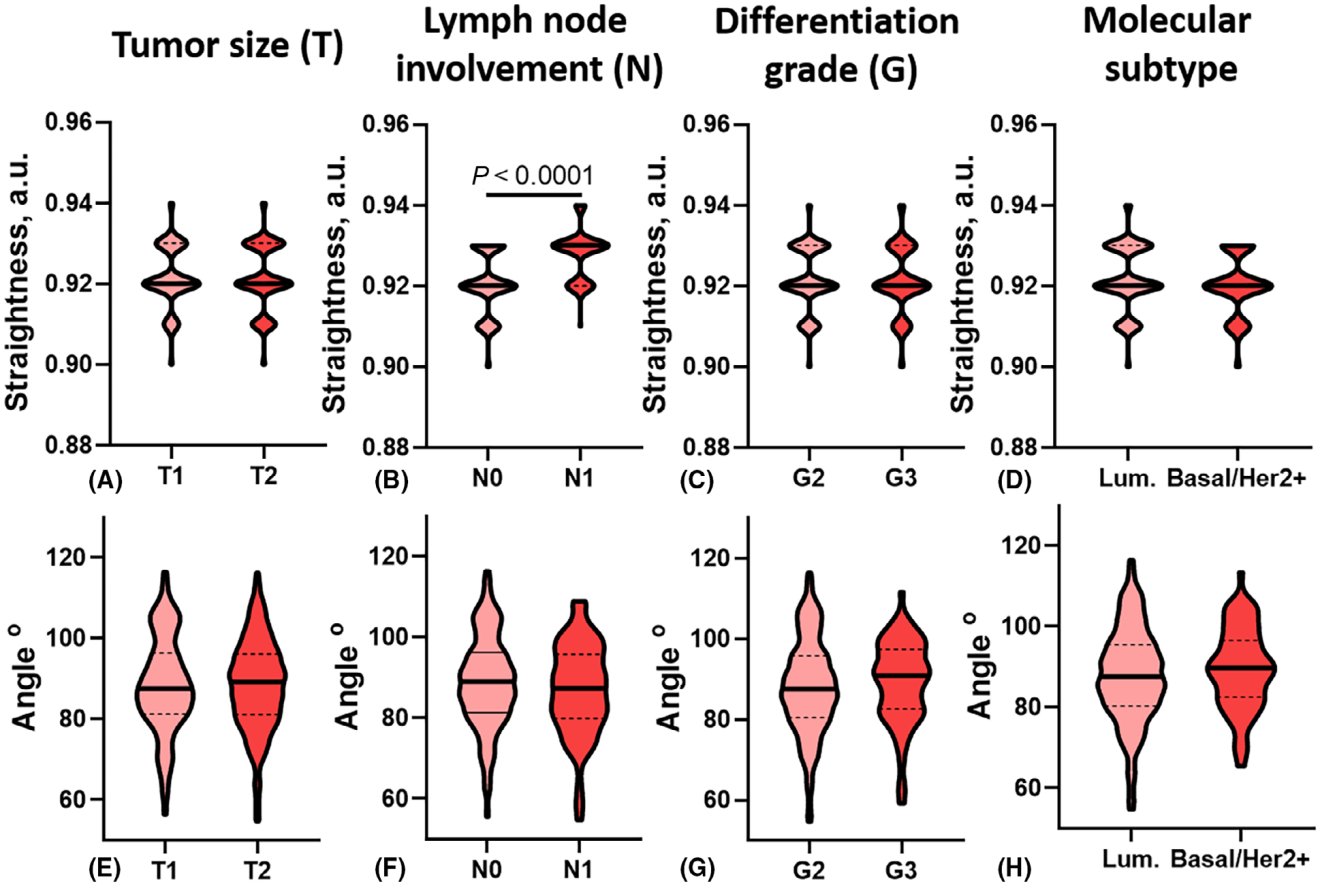
Quantitative indicators (A–D – straightness, E–H – angle) of the spatial organization of collagen in BCa tissue. Lum – luminal molecular subtype; Basal/Her2+ − Her2/neu positive or basal molecular subtype group.

As can be seen from Fig. 8, in patients with regional lymph node metastasis, collagen fibers in BCa tissue also had significantly higher straightness coefficient values (*P* < 0.0001). No relationship was found between the angle value of collagen fibers with the main clinical and pathological characteristics of BCa (Figs 7 and 8).

**Fig. 9:**
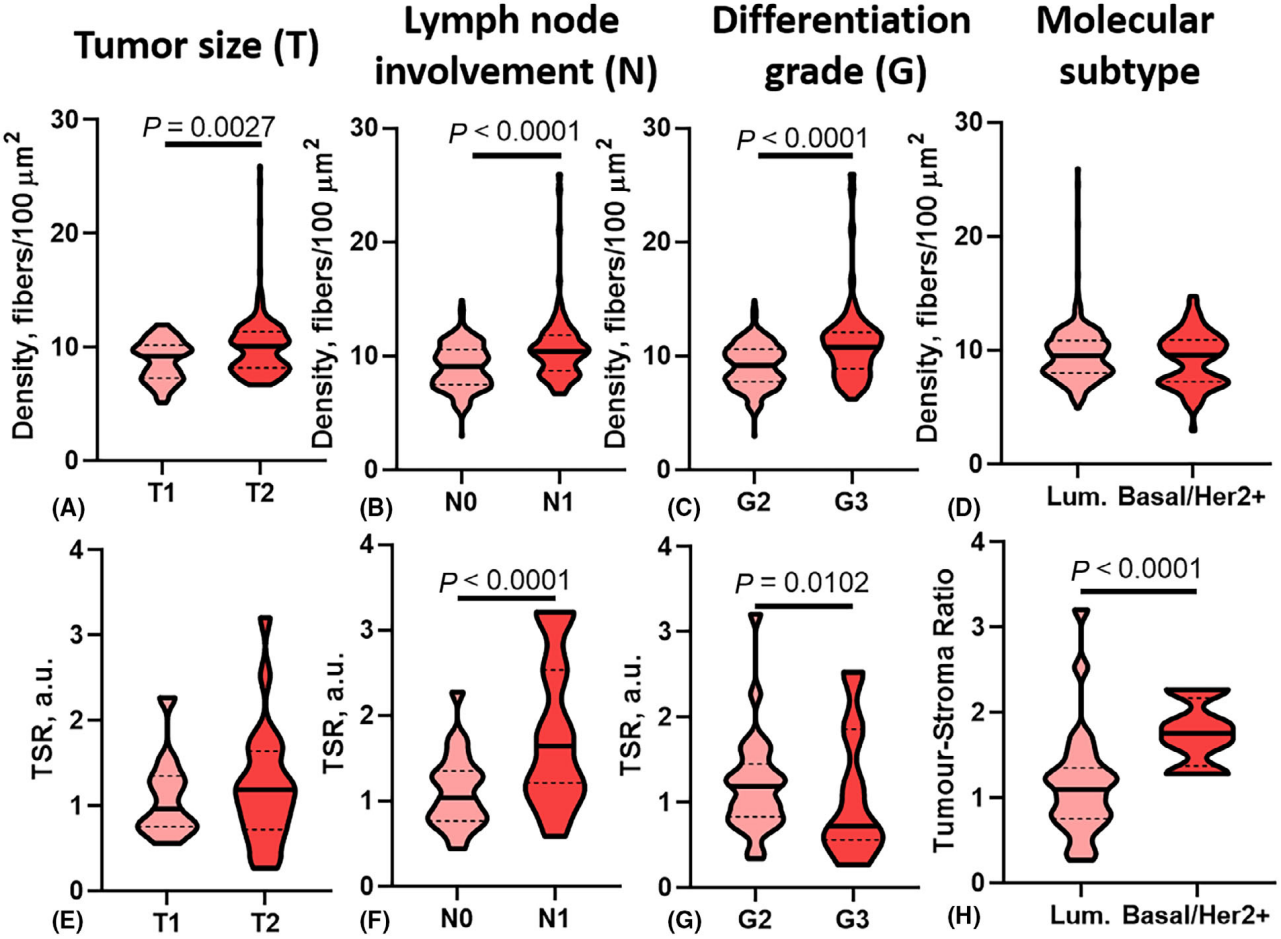
Quantitative indices (A–D – density, E–H – TSR) of the spatial organization of collagen in BCa tissue. Lum – luminal molecular subtype; Basal/Her2+ − Her2/neu positive or basal molecular subtype group.

A high density of collagen fibers was observed in BCa tissue of patients whose neoplasms were larger (*P* = 0.0027), in the cases of the presence of metastatic lesions in regional lymph nodes (*P* < 0.0001), and tumors of a low differentiation grade (*P* < 0.0001). TSR values were significantly higher in BCa tissue of the basal and HER2/neu positive molecular subtypes (*P* < 0.0001), compared to tumors of the luminal subtype. It was also shown, that TSR values had a direct relation to the lymph node involvement status (*P* < 0.0001) and invert relations to tumors differentiation grade (*P* = 0.0102) (Figs 8 and 9).

When analyzing the correlations between the clinical characteristics of BCa and the morphometric parameters of the collagen matrix spatial organization, the existence of an inverse relationship between the size of neoplasms and the collagen fibers width (*r* = −0.75101; *P* < 0.0001) and length (*r* = −0.65654; *P* < 0.0001) was revealed (*P* < 0.0001), as well as a direct correlation with the density of the collagen matrix (*r* = 0.491185; *P* = 0.00235). It is shown that the lymph node involvement is inversely correlated with the collagen fibers width (*r* = −0.71141; *P* < 0.0001), and also directly depends on TSR (*P* = 0.688217; *P* < 0.0001) and the density of the collagen matrix (*r* = 0.790265; *P* < 0.0001). It was found that an increase in the tumor grade is directly associated with the density of collagen fibers (*r* = 0.565149; *P* = 0.000329) and inversely correlated with the width of collagen fibers (*P* = −0.63849; < 0.0001). The existence of a direct correlation between the molecular subtype of BCa (*r* = 0.575725; *P* = 0.076934) and TSR indicators was noted (Table 5).

**Table 5:** Correlation matrix of clinical characteristics and collagen fibers quantitative indices.

	Tumor size (T)	Lymph node invilvment (N)	Differentiation grade (G)	Molecular subtype
Width	**−0.75101**	**−0.71141**	**−0.63849**	0.127689
Length	**−0.65654**	−0.15082	−0.05931	−0.20831
Straightness	0.2512	0.074914	0.069267	−0.14205
Angle	−0.18851	−0.1135	−0.02134	0.298565
Density	**0.491185**	**0.790265**	**0.565149**	−0.06608
TSR	−0.30372	**0.688217**	0.226493	0.575725

significant correlation is highlighted in bold.

Pearson correlation shown in below 
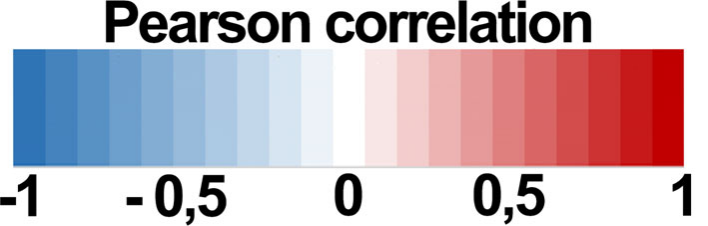

Taking into account the obtained data on the correlation of the tumoral collagen matrix morphometric parameters with such indicators of BCa malignancy as the size and degree of differentiation of neoplasms, as well as the lymph node involvement, we performed a predictive analysis of the dependence of clinical parameters and the collagen characteristics (Table 6).

**Table 6:** Results of multiple linear regression, where tumor size, lymph node involvement, and differentiation grade were determined as dependent variables.

		Tumor size (T)	Lymph node involvement (N)	Differentiation grade (G)
		*R* ^2^ = 0.67940939	*R* ^2^ = 0.77162898	*R* ^2^ = 0.48675879
Width	β	**−0.46332**	**−0.32793**	**−0.49441**
	*P*	**0.001399**	**0.012015**	**0.003810**
Length	β	**−0.32318**	0.112481	0.080845
	*P*	**0.014873**	0.250001	0.545608
Straightness	β	–	0.076409	–
	*P*	–	0.399713	–
Density	β	**0.242827**	**0.416609**	**0.327039**
	*P*	**0.032003**	**0.002070**	**0.041269**
TSR	β	–	**0.320168**	−0.0395
	*P*	–	**0.005705**	0.780628

significant models were highlighted in bold.

It was found that the width (*P* = 0.001399), length (*P* = 0.014873), and density of collagen fibers (*P* = 0.032003) significantly influence the size of the neoplasm. The coefficient of determination *R* ^2^ for such a model was 0.67940939. It was found that lymph node involvement depends on the width (*P* = 0.012015), density (*P* = 0.002070) of collagen fibers, and TSR (*P* = 0.005705). The resulting model had a coefficient of determination *R* ^2^ = 77 162 898. When predicting tumor grade depending on the morphometric parameters of the spatial organization of collagen, it was determined that only the width (*P* = 0.003810) and density of fibers (*P* = 0.041269) significantly influenced the BCa differentiation grade. The coefficient of determination for the obtained model was *R* ^2^ = 0.48675879.

## Discussion

So, an increased stage of the tumor process and a decreased tumor differentiation grade are associated with a decrease in the length, width, and straightness of collagen fibers, as well as with an increase in their density. The formation of an aggressive basal molecular BCa subtype is accompanied by an increase in TSR indices. The obtained data on the relationship of the above parameters of collagen fibers with the main clinical and pathological indices of BCa testify to the effectiveness of using the developed microphoto analysis algorithm to assess the spatial organization of the collagen fibrillar framework as part of the stromal microenvironment and coincide with the results of our previous studies [12]. Further study of the features of the spatial organization of collagen will allow us to identify the dynamical changes in its indices during cancer progression and to develop approaches to the personification of cancer therapy.

In recent years, the stromal microenvironment increasingly attracting the attention of researchers as one of the key and promising components of the tumor in the context of diagnosis and prediction of the course of breast cancer. At the same time, the morphometric parameters of collagen, which is the most common among the fibrillary proteins of the extracellular matrix, are associated with the tumor size and metastatic status of this form of cancer [8, 13].

Taking into account modern achievements in revealing the role of collagen as a biomarker of the initiation and progression of malignant neoplasms, an urgent task is the optimization of the methods for correctly determining not only the qualitative parameters of its structural organization but also the quantitative indicators of its spatial characteristics. For this purpose, for a detailed description of the spatial organization of the collagen fibrillary framework as part of the stromal microenvironment of the mammary gland malignant neoplasms, we have developed an algorithm for the analysis of microphotos using light microscopy methods, which can be used in routine practice.

The analysis of the spatial organization of collagen in the tumor tissue of patients with different clinical statuses confirmed the existing literature data regarding the high intensity of stroma remodeling processes during the progression of breast cancer. It was found that an increase in the density of fibers, as well as a decrease in their length and width, is associated with such indicators of the malignancy of breast cancer as stage T, the presence of metastasis in the regional lymph nodes, and the neoplasms grade. In our opinion, verified changes in the disposition of collagen fibers can be caused by the dysregulation of matrix metalloproteinases and osteopontin gene expression during the development and progression of the tumor process [14, 15, 16, 17].

In the literature available to us, there are only isolated reports regarding the association of TSR value with the molecular subtype of BC. Liu *et al*. and Yang *et al*. *in vitro* studies established that MDA‐231‐MB cells of the triple‐negative molecular subtype of breast cancer were characterized by high levels of expression of miR‐27a, mir‐145, and miR‐146a, which suppress the expression of some fibrillary‐type collagen genes. According to other researchers, the increase in TSR indicators in the tissue of the basal molecular subtype of breast cancer probably indicates the intensification of the tumor parenchyma growth processes, while the volume of the stromal component of neoplasms remains unchanged [18, 19, 20].

According to the obtained data, the increase in the straightness of collagen fibers in the tissue of patients with breast cancer was associated with the presence of metastatic lesions of regional lymph nodes. It has been proven that the length of fibers along which disseminated cells move is a key factor in the processes of migration and spread of transformed cells and tumor metastasis [21].

Thus, our results indicate the possibility of practical application of the developed algorithm for the evaluation of the spatial organization of collagen, as the main stromal component of the breast cancer microenvironment for predicting the aggressiveness of the course of the tumor process. Further research will improve the currently existing methods of analyzing microscopic images of histological slides for the fibrillary components morphology study in tumor tissue and will significantly improve the quality of differential diagnosis and prognosis of the malignant neoplasms course.

## Conflict of interest

All authors have no actual or potential conflict of interest including any financial, personal, or other relationships with other people or organizations within 3 years of beginning the submitted work that could inappropriately influence, or be perceived to influence their work.

### Peer review

The peer review history for this article is available at https://www.webofscience.com/api/gateway/wos/peer‐review/10.1002/2211‐5463.13773.

## Author contributions

Conception and Design: VC, NL; collection and assembly of data: TZ, OM, NL; data analysis and interpretation: TZ, OM, NL, VC; manuscript writing: OM, TZ, NL; final approval of manuscript: VC; agree to be accountable for all aspects of the work, which includes ensuring that questions related to the accuracy or integrity of any part of the work are appropriately investigated and resolved: OM, NL.

## Data Availability

The data described within this study can be provided by the corresponding author upon reasonable request.
